# Kikuchi Disease with Generalized Lymph Node, Spleen and Subcutaneous Involvement Detected by Fluorine-18-Fluorodeoxyglucose Positron Emission Tomography/Computed Tomography

**DOI:** 10.4274/mirt.25338

**Published:** 2016-06-06

**Authors:** Alshaima Alshammari, Evangelia Skoura, Nafisa Kazem, Rasha Ashkanani

**Affiliations:** 1 Mubarak Al Kabeer Hospital, Clinic of Nuclear Medicine, Jabriya, Kuwait; 2 University College London Hospital, Clinic of Nuclear Medicine, London, United Kingdom

**Keywords:** Kikuchi-Fujimoto disease, histiocytic necrotizing lymphadenitis, Fluorine-18-fluorodeoxyglucose

## Abstract

Kikuchi-Fujimoto disease, known as Kikuchi disease, is a rare benign and self-limiting disorder that typically affects the regional cervical lymph nodes. Generalized lymphadenopathy and extranodal involvement are rare. We report a rare case of a 19-year-old female with a history of persistent fever, nausea, and debilitating malaise. Fluorine-18-fluorodeoxyglucose positron emission tomography/computed tomography (18F-FDG PET/CT) revealed multiple hypermetabolic generalized lymph nodes in the cervical, mediastinum, axillary, abdomen and pelvic regions with diffuse spleen, diffuse thyroid gland, and focal parotid involvement, bilaterally. In addition, subcutaneous lesions were noted in the left upper paraspinal and occipital regions. An excisional lymph node biopsy guided by 18F-FDG PET/CT revealed the patient’s diagnosis as Kikuchi syndrome.

## Abstract (tr)

Kikuchi hastalığı olarak bilinen Kikuchi-Fujimoto hastalığı, genellikle bölgesel servikal lenf düğümlerini etkileyen, nadir görülen benign ve kendini sınırlayıcı bir hastalıktır. Yaygın lenfadenopati ve ekstranodal tutulum nadirdir. Bu yazıda sürekli ateş, bulantı ve halsizlik şikayetleri olan 19 yaşında bir kadın hasta sunulmaktadır. Flor-18-florodeoksiglukoz pozitron emisyon tomografisi/bilgisayarlı tomografi (18F-FDG PET/BT) yaygın dalak, tiroid bezi ve fokal parotis katılımı ile bilateral servikal, mediastinal, aksiller, abdominal ve pelvik bölgelerde hipermetabolik multipl lenf nodları saptadı. Buna ek olarak, sol üst paraspinal ve oksipital bölgelerde subkutan lezyonlar mevcuttu. 18F-FDG PET/BT rehberliğinde yapılan eksizyonel lenf nodu biyopsisi ile Kikuchi sendromu tanısı kondu.

## INTRODUCTION

Kikuchi-Fujimoto disease (KFD) also known as Kikuchi disease or histiocytic necrotizing lymphadenitis is a rare idiopathic and self-limiting disorder that typically affects the regional cervical lymph nodes (1). Generalized lymphadenopathy with involvement of mediastinal, peritoneal, and retroperitoneal lymph nodes, and extra-nodal disease is a rare occurrence (2,3,4). Age at presentation is usually below 40 years with early reports showing female preponderance (female/male ratio, 4:1), while more recent data indicate that the actual male to female ratio is closer to 1:1 (5,6,7). Most cases have been reported from East Asia (8,9). In rare occasions, the condition was reported in children (10). The exact pathogenesis is not completely understood, and viral and autoimmune pathogenesis have been speculated. Reports have suggested the combined immune response of T cells and histiocytes (particularly apoptotic CD8+ and CD123 plasmacytoid monocytes) against infectious agents, as a possible cause (11). An article suggested an association between Mycobacterium szulgai lymphadenitis and KFD based on coexisting characteristic histologic features of KFD in lymph nodes and a positive culture for Mycobacterium szulgai (12). It has also been linked to other autoimmune conditions regarding pathogenesis, like systemic lupus erythematosus (SLE), anti-phospholipid syndrome, polymyositis, systemic juvenile idiopathic arthritis, bilateral uveitis, arthritis and cutaneous necrotizing vasculitis (13). KFD almost always has a benign course and resolves in several weeks to months (14). Its treatment is largely supportive, mainly with anti-inflammatory and antimicrobial drugs; hence differentiating it from other more serious conditions is important to guide management (15,16,17).

## CASE REPORT

**Figure 1:**
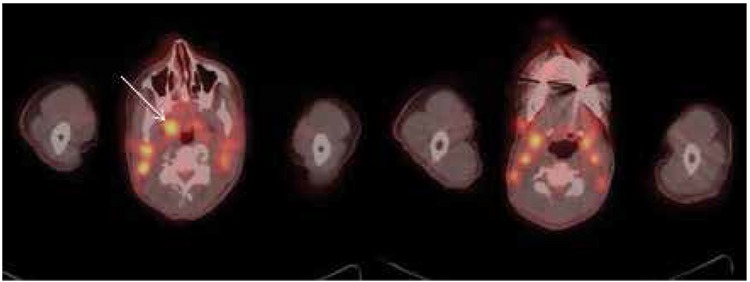
Increased fluorodeoxyglucose uptake in the deep cervical lymph nodes bilaterally, the largest and most avid node is noted in the right retropharyngeal region measuring 1.1x1.4 cm with SUVmax: 4.2 (arrow)

**Figure 2:**
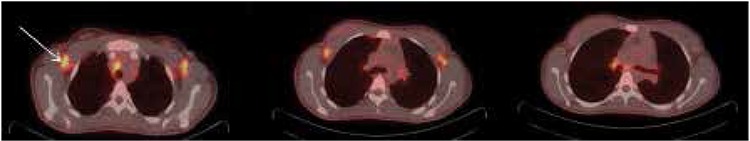
Increased fluorodeoxyglucose uptake in the pretracheal, subcarinal and right hilar lymph nodes and axillary lymph nodes bilaterally, most intense on the right axilla measuring 1.9x1.0 cm with SUVmax: 6.9 (arrow)

**Figure 3:**
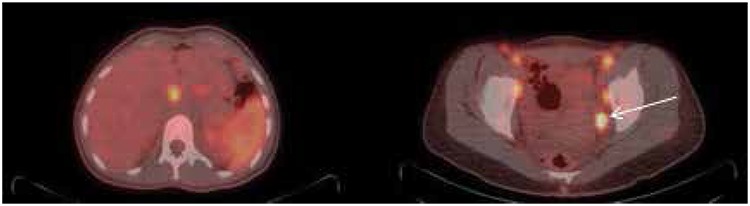
Increased fluorodeoxyglucose uptake in portocaval and external iliac chains bilaterally, most avid being the left external iliac lymph node measuring 1.7x1.5 with SUVmax: 7.5 (arrow), with associated diffuse splenic uptake

**Figure 4:**
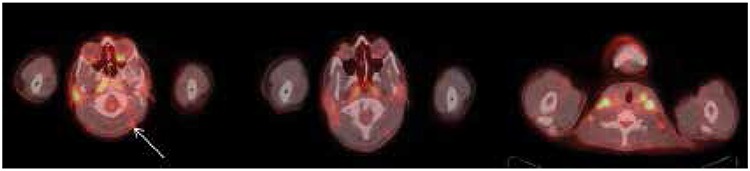
Subcutaneous mildly fluorodeoxyglucose avid lesions are seen in the occipital and upper para-spinal regions, SUVmax: 2.5 (arrow)

Apreviously healthy 19-years-old young woman presented with a history of persistent fever, nausea, debilitating malaise and bone pain. The patient had normal values of urea, creatinine, and serum electrolytes. She was investigated for SLE, but her antinuclear factor, double-stranded DNA, and anti-neutrophil cytoplasmic antibody were all negative. Blood and urine cultures were unremarkable. Viral serology for hepatitis and Epstein-Barr virus and Mantoux test were also negative. The patient underwent imaging with fluorine-18-fluorodeoxyglucose positron emission tomography/computed tomography (18F-FDG PET/CT) to investigate the cause of fever. 18F-FDG PET/CT scan showed multiple hypermetabolic lymph nodes with generalized involvement: in the neck (Figure 1), mediastinum (Figure 2), axillary (Figure 2), abdomen and pelvic regions with diffuse spleen uptake (Figure 3). In addition, hypermetabolic subcutaneous lesions in the left upper para-spinal and occipital regions were noted (Figure 4). Standardized uptake value (SUV) maximum standardized value (SUVmax) of 18F-FDG uptake in the affected lymph nodes and subcutaneous lesions was 6.3±2.4 (mean±SD), with lymph node size ranging from 0.7-1.9 cm in the long-axis diameter. The spleen was not enlarged measuring 10.7 cm in cranio-caudal dimension with a SUVmax value of 5.8. An excisional cervical lymph node biopsy guided by 18F-FDG PET/CT was performed. The histopathologic examination was consistent with the diagnosis of Kikuchi syndrome. Symptomatic treatment with antipyretics, non-steroidal anti-inflammatory drugs and low dose corticosteroids was administered. In the clinical follow-up after 3 months, she was symptom-free. No follow-up PET/CT study was performed.

## LITERATURE REVIEW AND DISCUSSION

KFD was first described in young Japanese females in 1972 (18). The patient’s usual presentation is tender regional cervical lymphadenopathy, sometimes associated with mild-grade fever (1). Only a few patients develop generalized lymphadenopathy and hepatosplenomegaly as the initial manifestations of KFD and even fewer cases are reported to have bone marrow and cutaneous, usually facial, involvement (3). The differential diagnoses of the condition include tuberculosis, and SLE. It can also mimic more serious conditions such as non-Hodgkin lymphoma (NHL), plasmacytoid T-cell leukemia, Kawasaki disease, nodal colonization by acute myeloid leukemia, and even metastatic adenocarcinoma (19). Multiple pathogens have been reported in isolated case reports such as Yersinia enterocolitica, Brucellosis, Bartonella henselae, Entamoeba histolytica, Mycobacterium szulgai, and Toxoplasma gondii, however, the fact that most patients with KFD are unresponsive to antibiotics suggests that these microbiologic organisms were incidental findings (12,17). The results of a wide range of laboratory studies are usually either normal or non-specific, such as anemia and slightly raised erythrocyte sedimentation rate (11). Recognition of KFD is crucial, especially since it can be mistaken for malignant lymphoma. A patient who has been misdiagnosed as having large-cell lymphoma and has been subjected to a course of cytotoxic therapy before submitting histologic sections to an expert pathologist has been previously reported (1). In fact, later studies suggested that up to 30% of patients with KFD have been reported to be initially misdiagnosed as malignant lymphoma and that some of them received unnecessary chemotherapy (20). KFD has been reported as one of the causes of prolonged fever of unknown origin (FUO). The utilization of 18F-FDG PET/CT in numerous clinical centers for finding the cause of fever in the diagnostic work-up of FUO is increasing. In general, causes of fever include malignant, infectious and non-infectious diseases (21). In their review article on the value of 18F-FDG PET and PET/CT in the diagnostic evaluation of patients with FUO, Meller et al. (22) found that FDG aided in reaching the final diagnosis with a frequency which varied between 25% and 69%. This article also demonstrated the wide range of possible causes of fever. In these studies, common causes of FUO detected by PET included various malignancies, several infectious diseases such as atypical pneumonia, spondylitis, tuberculosis, infected prostheses, and occult abscesses and non-infectious inflammatory diseases such as vasculitis, aortitis, and autoimmune diseases (22). PET imaging findings in KFD were first reported by Liao et al in 2003 (23). This was followed by multiple case reports and a few recent studies on 18F-FDG PET/CT trying to find distinguishing features between KFD and malignant lymph nodes (20). Ito and his group studied seven patients with KFD and found that the SUVmax values of 18F-FDG uptake in the affected lymph nodes were not beneficial for differentiating between benign and malignant tumors and that the values in the affected lymph nodes of patients with KFD were as high as the values found in malignancies (24). They suggested that the value of 18F-FDG PET/CT is that it can aid in excluding the metastatic involvement of extra-nodal sites in malignant lymphoma and help guiding decisions regarding appropriate biopsy sites (24). Similarly, in another study comparing clinical manifestations and PET/CT findings between KFD and lymphoma patients, Kim and his colleagues (25) found that there were no significant differences in SUVmax values between KFD and malignant lymphoma. They also concluded that increased uptakes in extra-nodal organs, such as bone marrow, small bowel, thymus, kidney, orbit, and pleura was the only distinguishing factor between lymphoma and KFD, but that only KFD with nodal involvement was indistinguishable from lymphoma. Another study suggested that in cases with a generalized distribution of small to medium-sized lymph nodes in 18F-FDG PET/CT with high 18F-FDG uptake, KFD should be considered as part of differential diagnosis (26). In another article concerning the value of 18F-FDG PET/CT in distinguishing KFD from NHL in patients with cervical lymphadenopathy, it was concluded that 18F-FDG PET/CT can be useful for distinguishing this disease from NHLs by using SUV and partial volume corrected SUV (cor SUV) (27).

## CONCLUSION

In conclusion, KFD is a rare, self-limited, and perhaps under-diagnosed condition. Recognition of this condition is crucial, especially because it can be mistaken for malignant lymphoma or adenocarcinoma. The awareness on this disease as a cause of fever and local lymphadenopathy, or rarely, as demonstrated in this case, generalized lymphadenopathy, might prevent misdiagnosis and inappropriate management. 18F-FDG PET/CT imaging may suggest the diagnosis of KFD, may depict the distribution and size of the affected lymph nodes, and guide an optimal lymph node biopsy.

## Ethics

Informed Consent: Consent form was filled out by all participants.

Peer-review: Externally peer-reviewed.

Financial Disclosure: The authors declared that this study has received no financial support.
